# Daily walking habits can mitigate age-related decline in static balance: a longitudinal study among aircraft assemblers

**DOI:** 10.1038/s41598-025-86514-w

**Published:** 2025-01-16

**Authors:** Kazuhiko Watanabe, Shoko Iizuka, Tatsuya Kobayashi, Saki Tsushima, Sora Hirohashi, Tomohiro Yoshimi, Masayoshi Zaitsu

**Affiliations:** 1 Center for Research of the Aging Workforce, University of Occupational and Environmental Health, Japan, Kitakyushu, Japan; 2 Kanagawa Health Service Association, Yokohama, Japan

**Keywords:** Age-related decline, Occupational fall, One-leg standing time, Static balance, Walking habits, Occupational health, Lifestyle modification, Preventive medicine, Rehabilitation

## Abstract

Improving physical balance among older workers is essential for preventing falls in workplace. We aimed to elucidate the age-related decline in one-leg standing time with eyes closed, an indicator of static balance, and mitigating influence of daily walking habits on this decline in Japan. This longitudinal study involved 249 manufacturing workers, including seven females, aged 20–66 years engaged in tasks performed at height in the aircraft and spacecraft machinery industry. The participants underwent a one-leg standing test and annual health checkups through the Kanagawa Health Service Association between 2017 and 2019. The outcome measure was one-leg standing time up to 30 s. The coefficient (β) of one-leg standing time against aging was estimated using two-level multilevel linear regression with random intercepts. We also estimated the β of daily walking habits at least one hour per day. The quadratic spline curve showed an almost linear trend of one-leg standing time with age. The one-leg standing time significantly decreased with age (adjusted β = − 0.22; 95% confidence interval [CI] − 0.31 to − 0.14). Meanwhile, walking habits showed a preventive effect (β = 1.76; 95% CI 0.49 to 3.04). Age-related decline in one-leg standing time may be mitigated by simple daily walking habits.

**Supplementary Information:**

The online version contains supplementary material available at 10.1038/s41598-025-86514-w.

## Introduction

With the aging of populations worldwide, older workers are increasingly being seen as an essential part of the labor workforce in Japan. For instance, while the number of people aged ≥ 65 years in Japan is approximately 36 million (28.8% of the population)^1^, the number of older workers aged ≥ 65 years has reached over 9 million (13.6% of the total workforce)^2^, and the employment rate of older workers was 25.2% in 2023^3^.

The incidence of occupational accidents, especially occupational fatalities and injuries, in this older working population has received attention, and effective prevention strategies are urgently needed. In Japan, 28.7% of the occupational accidents requiring a job leave of four days or more in 2022 occurred among older workers^4^. In particular, falls are the most common type of occupational accident in Japan, accounting for 28.8% of the total occupational accidents, and the incidence of occupational falls has been increasing^4^. In Japan, the number of fall-related occupational accidents requiring an absence of ≥ 4 days significantly increases from 40 years of age. The annual fall-related injury rate per 1,000 workers is lowest in the 25–29-years age group but rises progressively with age starting at 30 years^5^. Therefore, the risk of falls in middle and older ages can be alleviated by implementing measures aimed at maintaining physical function from a relatively young age.

For fall prevention in the workplace, occupational health strategies have mainly focused on improving environmental factors, including social management factors (e.g., organization and tidiness, haste and rule violations, and workplace culture) and external factors (e.g., floor friction, uneven surfaces, steps, handrails, lighting, and passage width). However, individual factors, including internal factors (e.g., motor and visual disabilities, physical and mental illnesses, and medication status) and injury-amplifying factors (e.g., physical strength and resilience, agility, bone strength, and internal organ resilience), have received less attention (Supplementary Fig. S1).

Static balance function is known to be an individual-level internal fall risk factor. Clinical and community-based studies have extensively used the one-leg standing test with eyes closed to evaluate static balance function^6–13^. Using this highly accurate and reproducible screening tool for fall risk, cross-sectional studies have reported shortening of one-leg standing time across different age categories, suggesting that static balance function declines with aging^14,15^. Among modifiable behavioral factors, walking has been suggested to improve static balance function^16^. However, longitudinal studies evaluating the temporal changes in this function are scarce. Moreover, the extent to which daily exercise habits, such as walking, improve this function has not been clarified.

We aimed to assess whether one-leg standing time with eyes closed, an indicator of static balance and occupational fall risk, declined with age in a working cohort in Japan. Additionally, we aimed to elucidate how daily walking habits may mitigate the age-related decline in one-leg standing time.

## Results

**Fig. 1:**
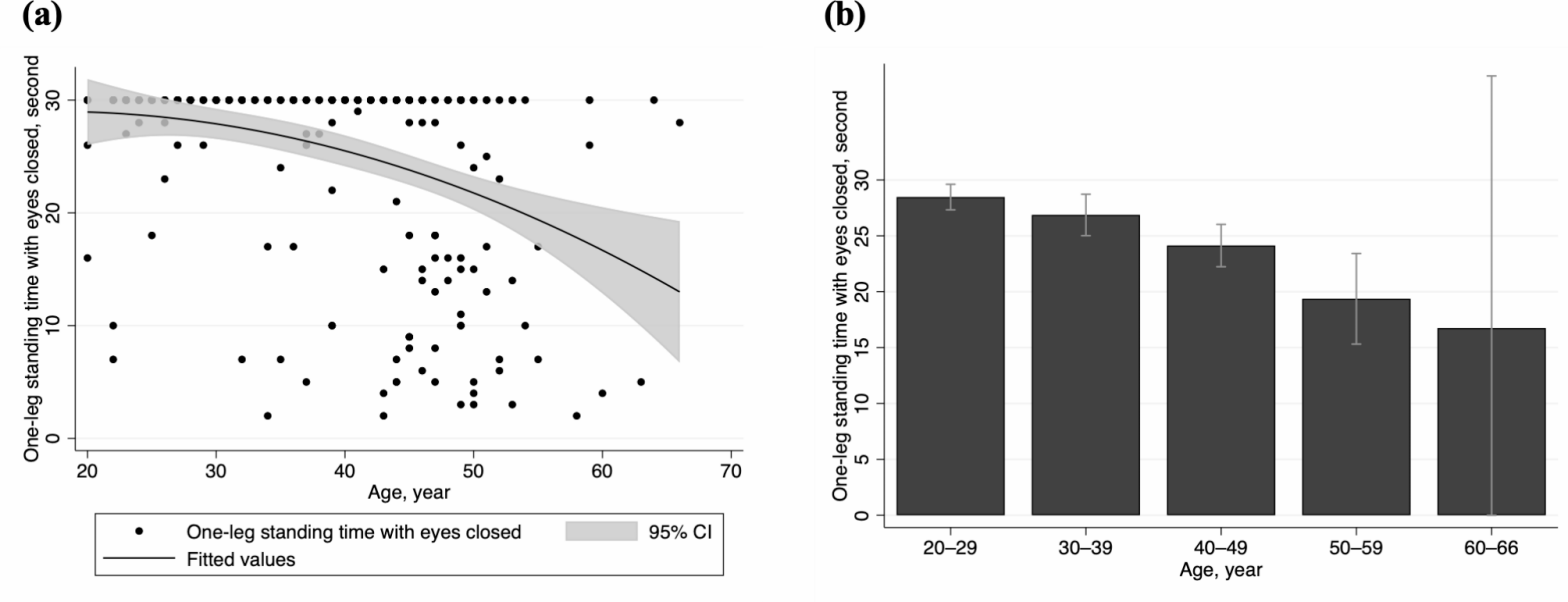
Distributions of the baseline one-leg standing time with eyes closed across 249 air and spacecraft manufacturing workers of different ages. (**a**) Scatter plots with a fitted quadratic spline curve illustrating individual data points and a linear declining trend across ages. The number of participants achieving 30 s of one-leg standing test was 51 (20–29 years), 47 (30–39 years), 59 (40–49 years), 12 (50–59 years), and 1 (60–66 years). (**b**) Bar charts representing the mean one-leg standing times for each 10-year age category, highlighting a declining trend in static physical balance by age (P for trend < 0.001). The mean (standard deviation) of one-leg standing time with eyes closed was 28.5 (4.5) s, 26.9 (7.2) s, 24.1 (9.1) s, 19.4 (10.9) s, and 16.8 (14.2) s at 20–29 years (*n* = 62), 30–39 years (*n* = 61), 40–49 years (*n* = 92), 50–59 years (*n* = 30), and 60–66 years (*n* = 4), respectively. The error bars in the graph represent the 95% confidence intervals.

The baseline characteristics are shown in Table 1. The mean (standard deviation [SD]) age and one-leg standing time with eyes closed were 38.6 (10.4) years and 25.2 (8.6) s, respectively. Among all participants, 65.5% reported having a daily walking habit, 52.6% smoked, and 21.3% consumed alcohol 4 or more days per week. Older workers had a shorter one-leg standing time (Supplementary Table S1), and the one-leg standing time decreased almost linearly with age in the cross-sectional association at baseline (Fig. 1).

**Table 1:** Baseline characteristics of the 249 study participants.

Characteristics	*N* (%) or mean (SD)
One-leg standing time with eyes closed, s, mean (SD)	25.2 (8.6)
Female	7 (2.8%)
Age, years, mean (SD)	38.6 (10.4)
Daily walking habit	163 (65.5%)
Cigarette smoking	
Never	118 (47.4%)
Former	44 (17.7%)
Current	87 (34.9%)
Alcohol drinking	
Never	61 (24.5%)
Former	8 (3.2%)
Sometimes (≤ 3 days/week)	127 (51.0%)
Often (4 or 5 days/week)	23 (9.2%)
Almost every day	30 (12.1%)
Body mass index, kg/m^2^, mean (SD)	23.0 (3.6)

SD, standard deviation.

In the longitudinal analysis employing two-level multilevel linear regression with all 575 data points (Table 2), a significant age-related decline in one-leg standing time was observed. After adjusting for sex, the age-related decline was β = – 0.29 (Model 1). Even after adjusting for modifiable behavioral factors in Model 2, this age-related decline remained statistically significant (β = – 0.22), indicating that each additional year of age was associated with a 0.22-s decrease in the one-leg standing time.

**Table 2:** Coefficients and 95% confidence intervals for one-leg standing time with eyes closed estimated using multilevel linear regression.

Variable	Coefficient and 95% confidence interval ^a^		
	Crude	Model 1 ^b^	Model 2 ^c^
Age	**– 0.29 (–0.37**,**– 0.21)**	**–0.29 (–0.37**,** − 0.20)**	**– 0.22 (– 0.31**,**– 0.14)**
Daily walking habit	**2.19 (0.88**,** 3.50)**		**1.76 (0.49**,** 3.04)**
Female	3.99 (– 1.74, 9.72)	1.34 (–4.01, 6.69)	0.65 (– 4.48, 5.79)
Smoking (vs. never smoker)			
Former smoker	**– 3.60 (– 6.15**,**– 1.06)**		– 1.10 (– 3.52, 1.32)
Current smoker	**– 2.11 (– 4.15**,**– 0.06)**		– 0.85 (– 2.73, 1.04)
Alcohol drinking (vs. never drinker)			
Former	0.51 (– 3.29, 4.31)		–0.55 (–4.15, 3.06)
Sometimes (≤ 3 days per week)	0.87 (– 1.20, 2.93)		0.42 (–1.52, 2.37)
Often (4, 5 days per week)	**– 3.24 (– 6.32**,**– 0.16)**		– 2.93 (– 5.91, 0.05)
Daily (almost everyday)	**– 4.53 (– 7.61**,**– 1.45)**		– 2.90 (– 5.86, 0.06)
Body mass index	**– 0.46 (– 0.71**,**– 0.21)**		**– 0.31 (– 0.54**,**– 0.08)**

^a^Coefficients were estimated using a two-level multilevel linear regression model. All 575 longitudinal data points of the one-leg standing time with eyes closed (Level 1) were nested within 249 individuals (Level 2), and a random intercept was employed for individuals. Bold face indicates *P* < 0.05.

^b^Adjusted for sex (confounder).

^c^Additional adjustments for modifiable behavioral factors (i.e., mediators), including daily walking habit, smoking, drinking, and body mass index.

In contrast, a daily walking habit was associated with an increase in one-leg standing time (β = 1.76 in Model 2, Table 2), suggesting that a daily walking habit may improve the one-leg standing time by 1.76 s. Sensitivity analyses showed a similar pattern as the main result (Table 3), and alcohol consumption and hearing loss also predicted a decline in one-leg standing time (Supplementary Table S2).

**Table 3:** Sensitivity analyses for age-related decline in one-leg standing time with eyes closed and beneficial impact of daily walking habits.

Variable	Coefficient and 95% confidence interval		
	Crude	Model 1 ^b^	Model 2 ^c^
143 participants who completed the one-leg standing test three times			
Age ^a^	**−0.28 (− 0.39**,** − 0.17)**	**−0.27 (− 0.38**,** − 0.17)**	**−0.22 (− 0.33**,** − 0.11)**
Daily walking habit ^a^	**2.23 (0.75**,** 3.70)**		**1.85 (0.39**,** 3.30)**
242 male participants			
Age ^a^	**−0.29 (− 0.37**,** − 0.20)**	**−0.29 (− 0.37**,** − 0.20)**	**−0.22 (− 0.31**,** − 0.14)**
Daily walking habit ^a^	**2.26 (0.91**,** 3.60)**		**1.80 (0.50**,** 3.11)**
249 initial data with a cross-sectional linear regression analysis			
Age	**−0.29 (− 0.38**,** − 0.19)**	**−0.28 (− 0.38**,** − 0.19)**	**−0.20 (− 0.30**,** − 0.10)**
Daily walking habit	**2.31 (0.07**,** 4.55)**		1.33 (− 0.72, 3.38)

^a^Coefficients were estimated using a two-level multilevel linear regression model. One-leg standing time data with eyes closed (Level 1) were nested within individuals (Level 2), and a random intercept was employed for individuals. Bold face indicates *P* < 0.05.

^b^Adjusted for sex (confounder).

^c^Additional adjustments for modifiable behavioral factors (i.e., mediators), including daily walking habit, smoking, drinking, and body mass index.

## Discussion

Using a longitudinal analysis design in a Japanese working cohort, we confirmed that the one-leg standing time with eyes closed, a physical indicator of static balance, declined linearly with age, decreasing by approximately 0.2 s per year starting in the early stages of working life. This suggests that the increase in occupational fall risk in the working lives of individuals may begin as early as in the 30s. We also found that daily walking habits may have beneficial effects on maintaining and/or improving static balance. Interestingly, the extent to which daily walking habits positively influenced one-leg standing time (approximately + 2 s) was approximately 10 times greater than the negative impact of aging (approximately − 0.2 s). While a decline in physical function at the workplace is a natural consequence of aging, our study revealed for the first time that adopting a habit of “walking” might improve the body’s static balance function.

Our results are consistent with previous findings showing that one-leg standing time and postural stability decline with age^14,17^. Three major sensory systems—visual, vestibular, and somatosensory—are crucial for maintaining balance, and reductions in one-leg standing time are influenced by age-related sensory declines^18^. A study of healthy men aged 30–80 years reported decreased postural stability and sensory function in their 60s^19^. The mean velocity of sway in a one-leg standing position with eyes closed was significantly lower in individuals in their 40s to 50s compared to those in their 30s^19^. Therefore, the one-leg standing balance test with eyes closed is useful to understand and monitor changes in physical function due to aging in working-age populations.

Our finding that daily walking habits may improve balance function is particularly important for workers engaged in high-place tasks, where falls from heights can lead to serious workplace injuries such as neurotrauma and fatalities. Although high levels of physical activity have been reported to help maintain postural stability^20^, a recent randomized controlled trial found that brisk walking improved one-leg standing balance in older women, consistent with our results^16^. Additionally, one-leg standing time has been associated with bone fractures^21,22^. Therefore, evaluating one-leg standing balance could serve as a screening tool for those with high risk of serious occupational injuries.

This study had several limitations that require consideration. First, the sample size was small, and the results from a single industry may have limited generalizability. Additionally, data on fall history or work-related injuries were not available, and the maximum one-leg standing time measurement was capped at 30 s, which may have introduced a ceiling effect. Future studies are needed to validate these associations using alternative assessment measures. Furthermore, healthy workers engaged in tasks at heights are likely to have good postural stability^23^, potentially underestimating the impact of age-related declines in static balance. However, one-leg standing is likely a robust indicator of fall risk^10,24^. Second, specific types of physical exercise and sports and intensity were not included^25^. Therefore, further research is needed to identify specific types of exercise and sports activities that may impact static balance function. Nevertheless, daily walking habits were shown to be beneficial for maintaining static balance. Additionally, other behavioral factors, including alcohol consumption and hearing loss, were found to be risk factors for declining static balance. Third, the impact of work experience on postural stability was not assessed^26^. Nevertheless, our results suggest that static balance tends to decline gradually in the early stages of an individual’s working life.

Despite these limitations, using a longitudinal design in a working cohort in Japan, this study is the first to simultaneously elucidate the specific deteriorating impact of aging and the preventive effects of daily walking habits on static balance in an aging workforce for whom fall prevention is a priority. Additionally, similar studies covering the aircraft manufacturing industry are scarce, and the data in the present study were obtained during annual health checkups to ensure validity.

In conclusion, the static balance of workers tends to begin declining linearly in the early stages of working life, while daily walking habits may offer a promising approach to mitigate the occupational fall risk in aging workplaces in Japan. Although national occupational health strategies have primarily focused on improving environmental factors, emphasizing individual factors, such as balance function, is also crucial. Thus, future studies should highlight nature-based strategies^27–30^ and encourage physical exercise in younger working populations to prevent fall-related occupational accidents.

## Methods

### Study design and study participants

This longitudinal study was conducted using data obtained from annual health checkups at the Kanagawa Health Service Association, Kanagawa, Japan. The participants were 249 workers (seven women, 2.8%) aged 20–66 years (mean age, 38.6 years; SD, 10.4 years), engaged in the manufacturing of aircraft, spacecraft, and related machinery at a single company. The participants underwent annual health checkups and additional measurements with the one-leg standing test with eyes closed. Between June 2017 and July 2019, 249 participants underwent a one-leg standing test at least once over 3 years. Although we intended to exclude participants with missing data for age, sex, or exercise habits, none of the participants met any of these criteria. All participants underwent health checkups performed by a physician and provided written informed consent.

This study was performed in accordance with the Declaration of Helsinki and approved by the Ethics Committee of the University of Occupational and Environmental Health, Japan (No. R4-054).

### Main outcome: one-leg standing time with eyes closed

The one-leg standing test with eyes closed was performed annually between 2017 and 2019. Of the 249 participants, 66 completed the test once, 40 completed it twice, and 143 completed it three times over the three-year period, yielding a total of 575 data points (218 in 2017, 183 in 2018, and 174 in 2019). Further details are provided in Supplementary Table S3.

The participants underwent the one-leg standing test with their eyes closed using a self-selected lower limb under the monitoring of a nurse. During the measurements, participants were instructed to keep their arms free, avoid touching the other leg, and keep their eyes closed^17^. The procedure was repeated twice, and the best (longer) of the two trials was recorded. The goal was to maintain balance for 30 s^17^. If the participants remained standing for 30 s, a time of 30 s was recorded. The investigator used a digital stopwatch and stopped the time measurement when either the raised foot touched the floor or when a maximum of 30 s had elapsed.

### Participants’ age, daily walking habits, and other characteristics

In addition to recording the participants’ age at the time of the health check-up, we assessed daily walking habits using the following question: “Do you engage in walking or physical activity equivalent to walking for at least 1 hour per day?” (yes/no).

Other characteristics included body mass index (BMI), alcohol consumption (none, former, sometimes [≤ 3 days per week], often [4 or 5 days per week], or daily [almost every day]), and smoking status (never, former, or current). The data were collected during health checkups (see full characteristics, including hearing loss, defined as abnormal findings in daily conversation at 1–4 kHz, in Supplementary Table S1).

### Statistical analysis

First, among the initial 249 measurement data points in the cross-sectional design, we identified an age-related linear declining trend in one-leg standing time with eyes closed using a quadratic spline curve. Additionally, differences in the mean one-leg standing time across 10-year age groups were described.

Next, using all 575 data points in a longitudinal design, we defined the causal pathway (Supplementary Fig. 2) as follows: age as the exposure, one-leg standing time with eyes closed as the outcome, and sex as a confounding factor in our regression analyses (Model 1). Model 2 included modifiable behavioral variables as mediating factors, including daily walking habits, smoking status, alcohol consumption, and BMI. To estimate the coefficient (β) and 95% confidence interval for one-leg standing time with eyes closed, we used a two-level multilevel linear regression model. All one-leg standing time data with eyes closed (Level 1) were nested within 249 individuals (Level 2), and a random intercept was employed for individuals.

For sensitivity analyses, we performed the same regression analysis among 143 participants who completed the one-leg standing test three times over three years. Additionally, we conducted a restricted analysis of male workers. A cross-sectional analysis was conducted using the initial 249 data points. Finally, we performed a linear regression analysis that included all potential covariates. Alpha was set at 0.05, and all p-values were two-sided. Data were analyzed using the STATA/MP17 software (StataCorp LLC, College Station, TX, USA).

## Electronic supplementary material

Below is the link to the electronic supplementary material.

Supplementary Material 1.

## Data Availability

The datasets used in the current study are available from the corresponding author upon reasonable request.

## References

[1] Ministry of Internal Affairs and Communications. Population estimates (Population estimates: confirmed Population as of the 1st of each Month [e-Stat Portal Site of Official Statistics of Japan, https://www.e-stat.go.jp/dbview?sid=0003443840])

[2] Ministry of Internal Affairs and Communications.: Labour Force Survey (Labour Force Survey Basic Tabulation Whole Japan Yearly, Population of 15 years old and over by labour force status and age groups [e-Stat Portal Site of Official Statistics of Japan, https://www.e-stat.go.jp/dbview?sid=0002060047])

[3] Ministry of Internal Affairs and Communications. : Labour Force Survey (Labour Force Survey Basic Tabulation Whole Japan Yearly, Labour force participation rate, employment rate and unemployment rate by age groups [e-Stat Portal Site of Official Statistics of Japan, https://www.e-stat.go.jp/dbview?sid=0002060049])

[4] Ministry of Health. Labour and Welfare. Labour Statistics. https://anzeninfo.mhlw.go.jp/user/anzen/tok/anst00.html

[5] Ministry of Health, Labour and Welfare. Analysis of occupational accident trends in 2023. Article in Japanese. (2024). https://www.mhlw.go.jp/content/11302000/001099504.pdf

[6] Vellas, B. J. et al. One-leg balance is an important predictor of injurious falls in older persons. *J. Am. Geriatr. Soc.* **45**, 735–738. 10.1111/j.1532-5415.1997.tb01479.x (1997). 9180669 10.1111/j.1532-5415.1997.tb01479.x

[7] Ek, S. et al. Predicting first-time injurious falls in older men and women living in the community: Development of the first injurious fall screening tool. *J. Am. Med. Dir. Assoc.* **20**, 1163–1168e3. 10.1016/j.jamda.2019.02.023 (2019). 30954420 10.1016/j.jamda.2019.02.023

[8] Rikkonen, T. et al. Long-term effects of functional impairment on fracture risk and mortality in postmenopausal women. *Osteoporos. Int.* **29**, 2111–2120. 10.1007/s00198-018-4588-4 (2018). 29860666 10.1007/s00198-018-4588-4

[9] Blain, H. et al. Self-reported fatigue: a significant risk factor for falling in older women and men. *Exp. Gerontol.* **143**, 111154. 10.1016/j.exger.2020.111154 (2021). 33189836 10.1016/j.exger.2020.111154

[10] Blodgett, J. M. et al. One-legged balance performance and fall risk in mid and later life: Longitudinal evidence from a British birth cohort. *Am. J. Prev. Med.* **63**, 997–1006. 10.1016/j.amepre.2022.07.002 (2022). 35995713 10.1016/j.amepre.2022.07.002 PMC10499759

[11] Blanco-Rambo, E. et al. Dance as an intervention to reduce fall risk in older adults: A systematic review with a meta-analysis. *J. Aging Phys. Act.* **30**, 1118–1132. 10.1123/japa.2021-0404 (2022). 35500909 10.1123/japa.2021-0404

[12] Wei, F., Hu, Z., He, R. & Wang, Y. Effects of balance training on balance and fall efficacy in patients with osteoporosis: A systematic review and meta-analysis with trial sequential analysis. *J. Rehabil Med.* **55**, jrm00390. 10.2340/jrm.v55.4529 (2023). 37194565 10.2340/jrm.v55.4529 PMC10348058

[13] Hurvitz, E. A., Richardson, J. K., Werner, R. A., Ruhl, A. M. & Dixon, M. R. Unipedal stance testing as an indicator of fall risk among older outpatients. *Arch. Phys. Med. Rehabil*. **81**, 587–591. 10.1016/s0003-9993(00)90039-x (2000). 10807096 10.1016/s0003-9993(00)90039-x

[14] Springer, B. A., Marin, R., Cyhan, T. & Roberts, H. Gill, N. W. Normative values for the unipedal stance test with eyes open and closed. *J. Geriatr. Phys. Ther.* **30**, 8–15. 10.1519/00139143-200704000-00003 (2007). 19839175 10.1519/00139143-200704000-00003

[15] Urabe, Y. et al. The difference in balance ability between generations. *Jpn J. Athl Train. (in Japanese)*. **5**, 133–139. 10.24692/jsatj.5.2_133 (2020).

[16] Sun, W. et al. Effects of Tai Chi Chuan and brisk walking exercise on balance ability in elderly women: a randomized controlled trial. *Mot. Control*. **23**, 100–114. 10.1123/mc.2017-0055 (2019). 10.1123/mc.2017-0055 30008242

[17] Bohannon, R. W., Larkin, P. A., Cook, A. C., Gear, J. & Singer, J. Decrease in timed balance test scores with aging. *Phys. Ther.* **64**, 1067–1070. 10.1093/ptj/64.7.1067 (1984). 6739548 10.1093/ptj/64.7.1067

[18] Winter, D. A. Human balance and posture control during standing and walking. *Gait Posture*. **3**, 193–214. 10.1016/0966-6362(96)82849-9 (1995).

[19] Illing, S., Choy, N. L., Nitz, J. & Nolan, M. Sensory system function and postural stability in men aged 30–80 years. *Aging Male*. **13**, 202–210. 10.3109/13685531003657826 (2010). 20201641 10.3109/13685531003657826

[20] Prioli, A. C., Freitas Júnior, P. B. & Barela, J. A. Physical activity and postural control in the elderly: Coupling between visual information and body sway. *Gerontology* **51**, 145–148. 10.1159/000083984 (2005). 15832038 10.1159/000083984

[21] Najafi, D. A., Dahlberg, L. E. & Hansson, E. E. A combination of clinical balance measures and FRAX^®^ to improve identification of high-risk fallers. *BMC Geriatr.* **16**, 94. 10.1186/s12877-016-0266-6 (2016). 27142632 10.1186/s12877-016-0266-6 PMC4855351

[22] Lim, Y., Ha, J., Yoon, K. H., Baek, K. H. & Kang, M. I. Measures of physical performance as a predictor of fracture risk independent of BMD: The Chungju metabolic disease cohort study. *Bone* **145**, 115878. 10.1016/j.bone.2021.115878 (2021). 33571697 10.1016/j.bone.2021.115878

[23] Cyma, M., Marciniak, K., Tomczak, M. & Stemplewski, R. Postural stability and physical activity of workers working at height. *Am. J. Mens Health*. **12**, 1068–1073. 10.1177/1557988318774996 (2018). 29790409 10.1177/1557988318774996 PMC6131451

[24] Welmer, A. K., Rizzuto, D., Laukka, E. J., Johnell, K. & Fratiglioni, L. Cognitive and physical function in relation to the risk of injurious falls in older adults: A population-based study. *J. Gerontol. Biol. Sci. Med. Sci.* **72**, 669–675. 10.1093/gerona/glw141 (2017). 10.1093/gerona/glw141 27449140

[25] Hahn, T., Foldspang, A., Vestergaard, E. & Ingemann-Hansen, T. One-leg standing balance and sports activity. *Scand. J. Med. Sci. Sports*. **9**, 15–18. 10.1111/j.1600-0838.1999.tb00201.x (1999). 9974192 10.1111/j.1600-0838.1999.tb00201.x

[26] Min, S. N., Kim, J. Y. & Parnianpour, M. The effects of safety handrails and the heights of scaffolds on the subjective and objective evaluation of postural stability and cardiovascular stress in novice and expert construction workers. *Appl. Ergon.* **43**, 574–581. 10.1016/j.apergo.2011.09.002 (2012). 21986560 10.1016/j.apergo.2011.09.002

[27] Struthers, N. A., Guluzade, N. A., Zecevic, A. A., Walton, D. M. & Gunz, A. Nature-based interventions for physical health conditions: A systematic review and meta-analysis. *Environ. Res.* **258**, 119421. 10.1016/j.envres.2024.119421 (2024). 38876421 10.1016/j.envres.2024.119421

[28] Sun, Y. et al. Association between urban green space and postpartum depression, and the role of physical activity: A retrospective cohort study in Southern California. *Lancet Reg. Health Am.* **21**, 100462. 10.1016/j.lana.2023.100462 (2023). 37223828 10.1016/j.lana.2023.100462 PMC10201204

[29] Garside, R., Lovell, R., Husk, K., Sowman, G. & Chapman, E. Nature Prescribing. *BMJ* **383**, 2745. 10.1136/bmj.p2745 (2023). 10.1136/bmj.p2745 38164636

[30] Coventry, P. A. et al. Nature-based outdoor activities for mental and physical health: Systematic review and meta-analysis. *SSM Popul. Health*. **16**, 100934. 10.1016/j.ssmph.2021.100934 (2021). 34646931 10.1016/j.ssmph.2021.100934 PMC8498096

