# Supplementary material for: Daily walking habits can mitigate age-related decline in static balance: a longitudinal study among aircraft assemblers
Source: Sci Rep. 2025 Jan 16;15:2207. doi: 10.1038/s41598-025-86514-w (PMC11742644; doi:10.1038/s41598-025-86514-w)

**Supplementary materials**

**Daily walking habits can mitigate age-related decline in static balance: a longitudinal study among aircraft assemblers**

Kazuhiko Watanabe^1^; Shoko Iizuka^2^; Tatsuya Kobayashi^2^; Saki Tsushima^1^; Sora Hirohashi^1^; Tomohiro Yoshimi^1^; Masayoshi Zaitsu^1^

^1^ Center for Research of the Aging Workforce,

University of Occupational and Environmental Health, Japan, Kitakyushu, Japan

^2^ Kanagawa Health Service Association, Yokohama, Japan

**Supplementary Table S1. Baseline characteristics of the study participants by different ages**

| Characteristics | Total | Age category | | P-value ^a^ |
| --- | --- | --- | --- | --- |
|  |  | 20–39 years | 40–66 years |  |
| N | 249 | 123 | 126 |  |
| One-leg standing time with eyes closed, sec^*^ | 25.2 (8.6) | 27.7 (6.0) | 22.8 (9.9) | <0.001 |
| Female | 7 (2.8%) | 7 (5.7%) | 0 (0.0%) | 0.007 |
| Age, years^*^ | 38.6 (10.4) | 29.7 (5.7) | 47.3 (5.2) | <0.001 |
| Daily walking habit | 163 (65.5%) | 85 (69.1%) | 78 (61.9%) | 0.232 |
| Cigarette smoking |  |  |  |  |
| Never | 118 (47.4%) | 66 (53.7%) | 52 (41.3%) | 0.045 |
| Former | 44 (17.7%) | 15 (12.2%) | 29 (23.0%) |  |
| Current | 87 (34.9%) | 42 (34.1%) | 45 (35.7%) |  |
| Alcohol drinking |  |  |  |  |
| Never | 61 (24.5%) | 30 (24.4%) | 31 (24.6%) | 0.006 |
| Former | 8 (3.2%) | 7 (5.7%) | 1 (0.8%) |  |
| Sometimes (≤3 days/week) | 127 (51.0%) | 69 (56.1%) | 58 (46.0%) |  |
| Often (4, 5 days/week) | 23 (9.2%) | 10 (8.1%) | 13 (10.3%) |  |
| Daily (almost every day) | 30 (12.1%) | 7 (5.7%) | 23 (18.3%) |  |
| Body mass index, kg/m^2*^ | 23.0 (3.6) | 22.4 (3.7) | 23.7 (3.4) | 0.002 |
| Body weight, kg^*^ | 67.7 (11.0) | 66.0 (11.9) | 69.3 (9.8) | 0.009 |
| Height, cm^*^ | 171.5 (6.1) | 171.7 (5.9) | 171.3 (6.2) | 0.605 |
| Hearing loss | 14 (5.6%) | 0 (0.0%) | 14 (11.1%) | <0.001 |
| Hypertension | 13 (5.2%) | 1 (0.8%) | 12 (9.5%) | 0.002 |
| Diabetes mellitus | 2 (0.8%) | 0 (0.0%) | 2 (1.6%) | 0.161 |
| Dyslipidemia | 13 (5.2%) | 0 (0.0%) | 13 (10.3%) | <0.001 |
| Grip strength, kg^*^ | 42.5 (6.2) | 42.2 (6.5) | 42.8 (5.9) | 0.448 |
| Waist circumference, cm^*^ | n=174  83.3 (10.3) | n=48  82.0 (11.8) | n=126  83.8 (9.7) | 0.309 |
| Hemoglobin A1c^*^ | n=248  5.5 (0.4) | n=123  5.3 (0.3) | n=125  5.6 (0.5) | <0.001 |
| Creatinine^*^ | n=248  0.9 (0.1) | n=123  0.9 (0.1) | n=125  0.9 (0.1) | 0.012 |

^*^Mean and standard deviation.

^a^P-values for t-test or Chi-square test.

^b^Hearing loss was defined as abnormal findings in either daily conversations or 1 kHz/4 kHz hearing.

^c^Grip strength was measured on both sides, and the maximum value from either side was used in the analysis.

**Supplementary Table S2. Sensitivity analysis of a two-level multilevel linear regression among 248 participants who completed all potential covariates**

| Variable | Coefficient and 95% confidence interval ^a^ | | |
| --- | --- | --- | --- |
|  | Crude | Sex & age adjusted | Full adjustment |
| Age | **–0.28 (–0.36, –0.20)** | **–0.28 (–0.36, –0.19)** | **–0.19 (–0.28, –0.10)** |
| Daily walking habit | **2.11 (0.79, 3.42)** |  | **1.62 (0.35, 2.90)** |
| Female | 3.91 (–1.75, 9.57) | 1.38 (–3.94, 6.70) | 1.68 (–3.81, 7.17) |
| Smoking (vs. never smoker) |  |  |  |
| Former smoker | **–3.57 (–6.09, –1.05)** |  | –0.99 (–3.36, 1.38) |
| Current smoker | –1.91 (–3.94, 0.12) |  | –0.63 (–2.51, 1.24) |
| Alcohol drinking (vs. never drinker) |  |  |  |
| Former | 0.52 (–3.27, 4.31) |  | –0.32 (–3.93, 3.29) |
| Sometimes (≤3 days per week) | 0.87 (–1.18, 2.93) |  | 0.35 (–1.57, 2.27) |
| Often (4, 5 days per week) | **–3.22 (–6.29, –0.15)** |  | **–3.28 (–6.25, –0.30)** |
| Daily (almost everyday) | **–4.05 (–7.15, –0.95)** |  | **–3.05 (–6.03, –0.07)** |
| Body mass index | **–0.48 (–0.73, –0.23)** |  | **–0.36 (–0.60, –0.12)** |
| Hearing loss | **–4.90 (–8.00, –1.80)** |  | **–3.36 (–6.32, –0.40)** |
| Hypertension | –2.92 (–6.80, 0.95) |  | 1.39 (–2.24, 5.02) |
| Diabetes mellites | –1.59 (–8.09, 4.91) |  | –0.32 (–6.89, 6.25) |
| Dyslipidemia | **–3.79 (–6.97, –0.61)** |  | –2.32 (–5.51, 0.87) |
| Grip strength | –0.02 (–0.15, 0.11) |  | 0.05 (–0.08, 0.19) |
| Hemoglobin A1c | –0.96 (–2.70, 0.78) |  | 0.59 (–1.23, 2.41) |
| Creatinine | –0.64 (–8.03, 6.75) |  | 1.02 (–6.08, 8.13) |

^a^A two-level multilevel linear regression model was performed with 573 data points from 248 participants who completed all potential covariates. One-leg standing time data with eyes closed (Level 1) were nested within individuals (Level 2), and a random intercept was employed for individuals. Bold face indicates P<0.05.

**Supplementary Table S3. Number of data in each year by number of times the participants underwent one-leg standing tests**

| **Number of times the participant underwent the tests** | Total | **Year** | | |
| --- | --- | --- | --- | --- |
|  |  | **2017** | **2018** | **2019** |
| Once, n = 66 ^a^ | 66 | 47 | 1 | 18 |
| 2 times, n = 40 ^b^ | 80 | 28 | 39 | 13 |
| 3 times, n = 143 ^a^ | 429 | 143 | 143 | 143 |
| **Total number of data** | **575** | **218** | **183** | **174** |

^a^Number of participants.

^b^Number of participants who underwent the one-leg standing test in 2017 and 2018, 2018 and 2019, and 2017 and 2019 were 27, 12, and 1, respectively.

**Figure legend**

**Supplementary Figure S1. Four risk factors for fall accidents at the workplace** Author's modification and illustration citing Kawagoe T. Japanese Journal of Fall Prevention. 2020;6(3):9-14 (in Japanese).

**Supplementary Figure S2. Causal pathway**

In our regression analyses, we defined the causal pathway as follows: age as the exposure, one-leg standing time with eyes closed as the outcome, and sex as a confounding factor (Model 1). In Model 2, we included modifiable behavioral variables as mediating factors, including daily walking habits, smoking status, alcohol consumption, and body mass index.


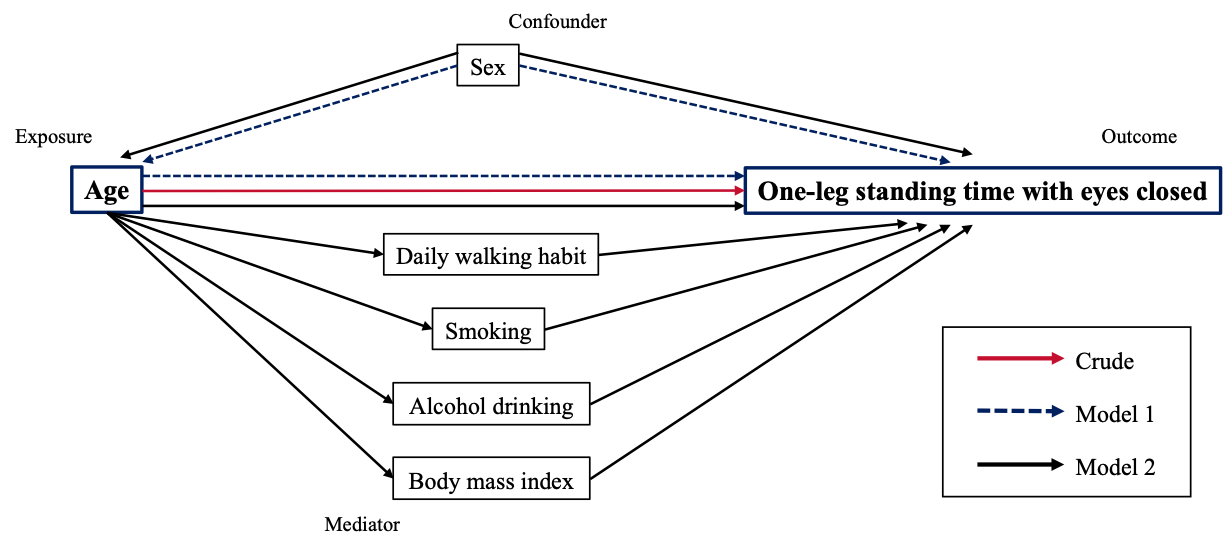

Supplement: Supplementary file 1 — Supplementary Material 1. [file 41598_2025_86514_MOESM1_ESM.docx]
